# The core inflammatory factors in patients with major depressive disorder: a network analysis

**DOI:** 10.3389/fpsyt.2023.1216583

**Published:** 2023-08-25

**Authors:** Yexian Zeng, Bin Sun, Fan Zhang, Zhibo Hu, Weicheng Li, Xiaofeng Lan, Yuping Ning, Yanling Zhou

**Affiliations:** ^1^Department of Child and Adolescent Psychiatry, Affiliated Brain Hospital of Guangzhou Medical University, Guangzhou, China; ^2^Guangdong Engineering Technology Research Center for Translational Medicine of Mental Disorders, Guangzhou, China; ^3^Key Laboratory of Neurogenetics and Channelopathies of Guangdong Province and the Ministry of Education of China Guangzhou Medical University, Guangzhou, China; ^4^Key Laboratory of Neurogenetics and Channelopathies of Guangdong Province and the Ministry of Education of China, The Second Affiliated Hospital, Guangzhou Medical University, Guangzhou, China; ^5^Department of Psychology,The First School of Clinical Medicine, Southern Medical University, Guangzhou, China

**Keywords:** network analysis, major depressive disorder (MDD), inflammatory factors, trait depression, cluster analysis

## Abstract

**Introduction:**

The symptoms of major depressive disorder (MDD) vary widely. Psycho-neuro-inflammation has shown that MDD’s inflammatory factors can accelerate or slow disease progression. This network analysis study examined the complex interactions between depressed symptoms and inflammatory factors in MDD prevention and treatment.

**Measures:**

We gathered participants’ inflammatory factor levels, used the Hamilton Depression Scale (HAMD-17), and network analysis was used to analyzed the data. Network analysis revealed the core inflammatory (nodes) and their interactions (edges). Stability and accuracy tests assessed these centrality measures’ network robustness. Cluster analysis was used to group persons with similar dimension depressive symptoms and examine their networks.

**Results:**

Interleukin-1β (IL-1β) is the core inflammatory factor in the overall sample, and IL-1β—interleukin-4 (IL-4) is the strongest correlation. Network precision and stability passed. Network analysis showed significant differences between Cluster 1 (with more severe anxiety/somatization and sleep disruption) and Cluster 3 (with more severe retardation and cognitive disorders), as well as between Cluster 2 (with more severe anxiety/somatization, sleep disruption and body weight) and Cluster 3. IL-1β is the core inflammatory factor in Cluster 1 and Cluster 2, while tumor necrosis factor alpha (TNF-α) in Cluster 3.

**Conclusion:**

IL-1β is the central inflammatory factor in the network, and there is heterogeneity in the core inflammatory factor of MDD with specific depressive dimension symptoms as the main manifestation. In conclusion, inflammatory factors and their links should be prioritized in future theoretical models of MDD and may provide new research targets for MDD intervention and treatment.

## 1. Introduction

Major depressive disorder (MDD) is a common mental illness. According to the latest World Health Organization (WHO), around 280 million people worldwide suffer from depression.^1^ According to the China Mental Health Survey, 95 million people in China suffer from depression, with a lifetime prevalence rate of 6.8% (1).

Inflammation has been linked to the pathophysiology of common adult psychiatric disorders, including major depressive disorder (2). Inflammatory markers in MDD patients have been studied more recently. An increasing number of studies have shown that depression is a systemic disease with multiple biological mechanisms, including the inflammatory response, hypothalamic-pituitary-adrenal (HPA) axonal dysregulation, and neurotransmitter and neurotrophic system imbalance. Stimulation of the hypothalamic-pituitary-adrenal axis (3) causes the release of tumor necrosis factor-α (TNF-α) and interleukin (IL) by proinflammatory cytokines (4). With the advancement of such research, the psycho-neuro-inflammatory theory has grown significantly, and numerous studies have revealed a close relationship between depressive symptoms and the expression of proinflammatory factors. Studies have shown that elevations in peripheral blood and central nervous system in proinflammatory cytokines, such as interleukin-1β (IL-1β), interleukin-6 (IL-6), interleukin-4 (IL-4), TNF-α, and interferon-γ (IFN-γ), can worsen depressive symptoms (5). These findings imply that inflammatory factors are important mediators of depression and play a role in the pathogenesis of MDD.

MDD has a wide range of symptoms that include somatic, motor, cognitive, and behavioral symptoms in addition to its core affective symptoms. There is evidence that the a specific subset of depressive symptom associates with specific inflammatory factor (6–11), and the association of inflammation with specific depressive symptoms may be clinically significant. IL-6 has been reported to be associated with increased appetite, sleep disorders, and mood swings during the day. Similarly, IL-1β, IL-6, and TNF levels in patients with MDD have been positively correlated with suicidal thoughts (12).

Depression is a phenotypically heterogeneous syndrome, and although there is clear evidence supporting the role of inflammation in the pathophysiology of depression (13–15), little is known about the core inflammatory markers associated with major depressive disorder (MDD). In addition, the connection between inflammatory factors and various dimensions of depressive symptoms is not fully understood. There has been little research on the interplay of inflammatory factors in patients with MDD. Furthermore, to the best of our knowledge, only a few studies have investigated the relationship between inflammatory markers and specific symptoms or dimensions of depression.

Previous studies (16, 17) have suggested that network analysis provides a novel approach to understanding the structure and interactions of various symptoms in mental disorders. This method involves constructing a network where symptoms are presented as nodes (16, 18). By analyzing central indicators of the network (betweenness, closeness, and strength), researchers can identify the core symptoms in the network structure and explore the connections between the symptoms. Various mental disorders, including, depression (19), anxiety (20), and other conditions have been studied using network analysis.

In this study, we conducted assessments using the 17-item Hamilton Depression Scale (HAMD-17) (21) and tested inflammatory factors in a large sample of patients diagnosed with MDD. This study aimed to (1) identify the most significant core inflammatory factors in patients with MDD and explore the relationships among inflammatory factors that had the greatest impact on the network and (2) compare the networks of inflammatory factors in MDD across various dimensions. This study holds the potential to pave the way for future investigations in neuro-inflammatory research on MDD by identifying possible inflammatory markers associated with the disorder. In addition, novel avenues and approaches for the clinical diagnosis and treatment of MDD were identified by investigating the connections between depressive symptoms along multiple dimensions, and inflammatory factors.

## 2. Methods

### 2.1. Participants

Data were collected from 280 outpatients with MDD who sought treatment at the Affiliated Brain Hospital of Guangzhou Medical University. The data were obtained from the baseline information of a larger study that aimed to investigate the molecular biomarkers associated with the response to treatment using antidepressants. All participants had a primary diagnosis of MDD between December 2016 and December 2019, according to the criteria outlined in the Diagnostic and Statistical Manual of Mental Disorders 5 (22). The study included patients who were in the acute phase (HAMD-17 total score >17). Patients younger than 18 and older than 65 years were excluded from the study. In addition, patients with severe comorbid psychotic disorders, substance addictions, those taking anti-inflammatory medications, pregnant women, and those with unstable medical conditions were excluded.

Prior to participating, all patients completed an informed consent form. The study was authorized by the Institutional Ethics Committee at the Affiliated Brain Hospital of Guangzhou Medical University and carried out in accordance with the Declaration of Helsinki (registration number: ChiCTR1800017626).

### 2.2. Clinical assessment

Depressive symptoms were assessed using the HAMD-17 scale, which is one of the most popular scales for evaluating depressive symptoms in clinical settings. This scale consists of five factors that encompass different aspects of depressive symptoms, as follows: (1) anxiety/somatization (i.e., mental anxiety, somatic anxiety, gastrointestinal symptoms, systemic symptoms, hypochondriasis, insight); (2) body weight (i.e., weight loss); (3) cognitive disruption (i.e., guilt, suicidal thoughts, agitation); (4) retardation (i.e., reduced work/interest, retardancy, sexual symptoms); and (5) sleep disruption (i.e., difficulty falling asleep, not sleeping deeply, waking up early) (23). The factor scores obtained from this assessment provide insights into the multidimensional distribution of clinical characteristics and symptoms associated with the disorder.

### 2.3. Assessment of inflammation

On the day of the commencement of the study, blood samples were collected from each participant. These samples were collected in EDTA-coated tubes and centrifuged at 3000 rpm for 10 min at 4°C. After centrifugation, plasma was extracted, transferred to Eppendorf tubes in measured aliquots, and stored at −80°C in a refrigerator. This ensured preservation and facilitated subsequent batch analysis of cytokines, namely IFN-γ, IL-1β, IL-4, IL-6, and TNF-α. To assess the concentrations of these cytokines, a high-human-sensitivity multiplex magnetic bead-based assay kit (HSTCMAG-28SK; Millipore, Billerica, MA, United States) was used. Milliplex Analyst 5.1 software was used to evaluate the data obtained from the measurement of cytokine concentrations. This software used a five-parameter logistic curve-fitting method to analyze the data and derive meaningful results from the concentrations of the five cytokines.

### 2.4. Statistical analysis

#### 2.4.1. Data analysis

SPSS Version 25.0 (IBM, Armonk, NY, United States) was used for data entry and statistical analysis. Initially, the mean and standard deviation of the demographic data, clinical data, and inflammatory factors were calculated for all patients diagnosed with MDD. Subgroups within the MDD group also received the same treatment, and potential clinical differences between these subgroups were evaluated using ANOVA and *post hoc* analysis.

#### 2.4.2. Cluster analysis

Clustering is a multivariate analysis technique used to classify items based on shared characteristics. Hierarchical clustering, *k*-means clustering, model-based clustering, and density-based clustering are the four most commonly used models for clustering analysis. The key benefits of model-based clustering methods are in terms of recommending the number of groups and determining the appropriate model, as opposed to the traditional clustering methods such as *k*-means clustering and hierarchical clustering (24) In our study, we used model-based cluster analysis (MBCA) using the R package mclust, version 6.0.0, to categorize patients into groups based on the level of correlation between their five depressive symptom variables. MBCA assumes that the observed data are derived from a distribution that combines with the Gaussian finite mixture model. To estimate the model parameters, we utilized the expectation–maximization algorithm to infer the best feasible model parameters and the Bayesian information criterion (BIC) to evaluate their appropriateness (24, 25). According to previous studies (26), the larger the value of the BIC, the stronger the evidence for the model and the number of clusters.

#### 2.4.3. Network estimation

This research network was constructed using the R package qgraph (version 1.9.3) (27). The Gaussian graph model (GGM) was chosen as the prediction model because it is appropriate for the study’s continuous data. Each inflammatory factor in the model is depicted as a “node” in the network, with the relationship between them being represented as an “edge” (17, 28). When the network is complete, this research reduced the number of unnecessary connections using a graphical LASSO algorithm (minimum contraction and selection operator) (29). Alternatively, the best model can be chosen using the extended Bayesian information standard (EBIC) (30) in a graphical representation of a network.

#### 2.4.4. Network centrality

To highlight which inflammatory factors may have the greatest impact on the depression network, we examined three major central indicators (i.e., betweenness, closeness, and strength) (31–34) and expected influence by the R package qgraph (version 1.9.3) (27), and the indices were shown as standardized *z* scores (35). Betweenness describes the extent to which a node is crossed by the maximum short distance between many nodes, closeness refers to the sum of the distances from a node to all points, strength refers to the sum of the weighted values of a node’s connections to other nodes, and finally, expected influence refers to the sign that retains the edge weights until the edge weights are summed (36, 37). In summary, the structural importance of each node in a network can be determined using node centrality (16, 36).

#### 2.4.5. Network accuracy and stability

To ensure the reliability of the networks, this research utilized the R package bootnet (1,000 times). First, the nodes were bootstrapped to ensure the consistency of the center of strength. Second, the 95% bootstrap confidence interval (CI) estimation bootstrapped difference test accuracy of the estimated network edge and centrality indices were analyzed (29). Third, the correlation stability coefficient (CS coefficient) was computed to quantify the subset-guided results (29). For a network to be considered stable, the CS coefficient of its centrality score must be greater than 0.25 (29).

#### 2.4.6. Network comparison test

The R package NetworkComparisonTest (version 2 2.1) (38) and qgraph (version 1.9.3) (27) were used to compare various sets of networks to investigate network differences in clusters. We conducted control measures for potential confounding variables before evaluating network differences between clusters. All inflammatory factors were regressed independently and then transformed into standardized residuals. Next, we established the cluster networks using the inflammatory factor residual variables as nodes and evaluated the differences in network characteristics between clusters. Variations in network structures were determined by calculating quantitative indicators in three areas: invariance of network structure, edge strength, and global strength (36, 38). We then compared the differences in network structure between the clusters after controlling for multiple comparisons using the Bonferroni correction. The statistical threshold for significance in a substitution process (1,000 repetitions) was set at *p* < 0.05.

## 3. Results

### 3.1. Statistics for study variables both overall and by cluster analysis

This study included 280 patients with MDD, whose mean age, education, BMI, and illness duration were 33.32 (11.80) years, 12.46 (3.20) years, 21.79 (3.75) kg/m^2^, and 30.79 (57.95) months, respectively. Among the patients, 55.70% were female and 51.80% were married. In addition, 79.30% had experienced their first MDD episode. The average score on the HAMD-17 scale was 23.40 ± 4.76. The mean scores for anxiety/somatization, retardation, cognitive disruption, sleep disruption, and body weight were 7.93 (2.33), 8.00 (1.92), 3.56 (1.91), 3.31 (1.96), and 0.69 (0.75), respectively. The average levels of IFN-γ, IL-1β, IL-4, IL-6, and TNF-α were 18.85 (14.42) pg/mL, 2.73 (2.16) pg/mL, 82.14 (89.49) pg/mL, 3.44 (5.85) pg/mL, and 6.63 (3.80) pg/mL, respectively (Table 1). As shown in Supplementary material S1, according to the results of the best model clustering calculation (BIC = −5290.179), the participants were grouped into three clusters: Cluster 1 (*n* = 91), Cluster 2 (*n* = 124), and Cluster 3 (*n* = 65).

**Table 1:** Demographic and clinical information for the overall sample.

Variables	Mean ± SD or *n* (%)
Age (years)	33.22 ± 11.80
BMI (kg/m^2^)	21.79 ± 3.75
Female	156 (55.7)
Married	145 (51.8)
Education years	12.46 ± 3.20
Illness duration (months)	30.79 ± 57.95
First episode	222 (79.3)
Antipsychotic drugs	44 (15.7)
IFN-γ	18.85 ± 14.42
IL-1β	2.73 ± 2.16
IL-4	82.14 ± 89.49
IL-6	3.44 ± 5.85
TNF-α	6.63 ± 3.80
AS	7.93 ± 2.33
R	8.00 ± 1.92
CD	3.56 ± 1.91
SLD	3.31 ± 1.96
W	0.69 ± 0.75
HAMD-17	23.40 ± 4.76

All concentrations are in pg/mL, IFN-γ, interferon gamma; IL, interleukin; TNF-α, tumor necrosis factor alpha; BMI, body mass index; AS, anxiety/somatization; R, retardation; CD, cognitive disturbance; SLD, sleep disruption; W, body weight; HAMD-17, 17-item Hamilton Depression Inventory.

The results of ANOVA for the demographic variables in this study were used to adjust for their possible confounding effects. As shown in Table 2, age (years), education (years), BMI (kg/m^2^), and illness duration (months) were used as continuous variables, whereas gender, first episode, antipsychotic drugs, and marital status were used as categorical variables. Based on ANOVA results, age, gender, marital status, and education did not show significant differences across the three clusters. However, substantial differences were observed in the illness duration, BMI, first episode of MDD, and use of antipsychotic drugs among the clusters; hence, these factors were considered significant confounders. Consequently, these variables were included as covariates, and the Bonferroni correction was applied to account for multiple comparisons. The corrected results are displayed in Table 3.

**Table 2:** Comparison of demographics, depressive symptom and plasma cytokines levels of participants between three clusters.

Variables	Cluster1 (*N* = 91)	Cluster2 (*N* = 124)	Cluster3 (*N* = 65)	*F*/*χ*^2^	*η* ^2^	Overall *p*-values	Cluster 1 vs. 2: *p*-values	Cluster 1 vs. 3: *p*-values	Cluster 2 vs. 3: *p*-values
Age (years)	34.86 ± 12.45	33.25 ± 12.20	30.88 ± 9.55	2.178	0.015	0.115			
BMI (kg/m^2^)	22.39 ± 3.85	20.97 ± 3.49	22.51 ± 3.85	5.506	0.038	0.005	0.016	1.000	0.021
Female	45 (28.8)	36 (23.1)	75 (48.1)	2.593		0.273			
Married	53 (36.6)	30 (20.7)	62 (42.8)	2.503		0.286			
Education years	12.03 ± 3.20	12.44 ± 3.33	13.10 ± 2.89	2.031	0.014	0.133			
Illness duration (months)	26.63 ± 56.26	22.62 ± 44.99	52.89 ± 75.07	6.663	0.045	0.002	1.000	0.014	0.002
First episode	76 (34.2)	106 (47.7)	40 (18.0)	16.358		<0.001		<0.05	<0.05
Antipsychotic drugs	12 (27.3)	20 (45.5)	12 (27.3)	14.974		<0.001		<0.05	<0.05
IFN-γ	19.42 ± 13.98	19.79 ± 14.89	16.27 ± 14.03	1.379	0.010	0.253			
IL-1β	2.70 ± 2.11	2.93 ± 2.26	2.40 ± 2.03	0.280	0.009	0.280			
IL-4	81.96 ± 72.49	85.05 ± 83.88	76.86 ± 118.13	0.837	0.001	0.837			
IL-6	2.71 ± 3.11	3.74 ± 5.19	3.88 ± 9.10	1.050	0.008	0.351			
TNF-α	6.63 ± 3.41	7.02 ± 4.15	5.88 ± 3.56	1.942	0.014	0.145			
AS	8.49 ± 2.54	8.10 ± 2.27	6.82 ± 1.70	11.220	0.075	<0.001	0.602	<0.001	<0.001
R	7.25 ± 1.72	8.29 ± 2.05	8.48 ± 1.61	11.122	0.074	<0.001	<0.001	<0.001	1.000
CD	3.08 ± 1.90	3.77 ± 1.95	3.85 ± 1.73	4.545	0.032	0.011	0.023	0.037	1.000
SLD	4.14 ± 1.47	3.80 ± 1.87	1.20 ± 1.03	76.527	0.356	<0.001	0.346	<0.001	<0.001
W	0.00 ± 0.00	1.35 ± 0.48	0.00 ± 0.00	618.717	0.817	<0.001	<0.001	1.000	<0.001
HAMD-17	22.97 ± 4.45	25.31 ± 4.94	20.34 ± 2.70	28.533	0.171	<0.001	<0.001	0.001	<0.001

All concentrations are in pg/mL, IFN-γ, interferon gamma; IL, interleukin; TNF-α, tumor necrosis factor alpha; BMI, body mass index; AS, anxiety/somatization; R, retardation; CD, cognitive disturbance; SLD, sleep disruption; W, body weight; HAMD-17, 17-item Hamilton Depression Inventory.

**Table 3:** Adjusted Comparison of depressive symptom and plasma cytokines levels of participants between three clusters.

Variables	Cluster1 (*N* = 91)	Cluster2 (*N* = 124)	Cluster3 (*N* = 65)	*F*	*η* ^2^	Overall *p*-values	Cluster 1 vs. 2: *p*-values	Cluster 1 vs. 3: *p*-values	Cluster 2 vs. 3: *p*-values
IFN-γ	19.07 ± 1.49	19.12 ± 1.29	18.03 ± 1.81	0.132	0.001	0.876			
IL-1β	2.65 ± 0.23	2.91 ± 0.20	2.52 ± 0.28	0.711	0.005	0.492			
IL-4	80.96 ± 9.29	80.15 ± 8.05	87.63 ± 11.28	0.152	0.001	0.859			
IL-6	2.63 ± 0.62	3.67 ± 0.54	4.13 ± 0.75	1.376	0.010	0.254			
TNF-α	6.49 ± 0.39	6.88 ± 0.34	6.35 ± 0.47	0.495	0.004	0.610			
AS	8.51 ± 0.24	8.05 ± 0.21	6.88 ± 0.29	9.673	0.066	<0.001	0.429	<0.001	0.004
R	7.29 ± 0.18	8.45 ± 0.16	8.12 ± 0.22	11.786	0.079	<0.001	<0.001	0.013	0.709
CD	3.09 ± 0.20	3.76 ± 0.17	3.87 ± 0.24	4.296	0.031	0.015	0.036	0.040	1.000
SLD	4.10 ± 0.17	3.78 ± 0.14	1.28 ± 0.20	65.742	0.325	<0.001	0.447	<0.001	<0.001
W	0.003 ± 0.03	1.36 ± 0.03	−0.008 ± 0.04	581.305	0.810	<0.001	<0.001	1.000	<0.001

All concentrations are in pg/mL, IFN-γ, interferon gamma; IL, interleukin; TNF-α, tumor necrosis factor alpha; BMI, body mass index; AS, anxiety/somatization; R, retardation; CD, cognitive disturbance; SLD, sleep disruption; W, body weight; HAMD-17, 17-item Hamilton Depression Inventory. Statistically significant after Bonferroni correction. Considering BMI, illness duration, first episode, and antipsychotic drugs as covariates.

**Figure 1:**
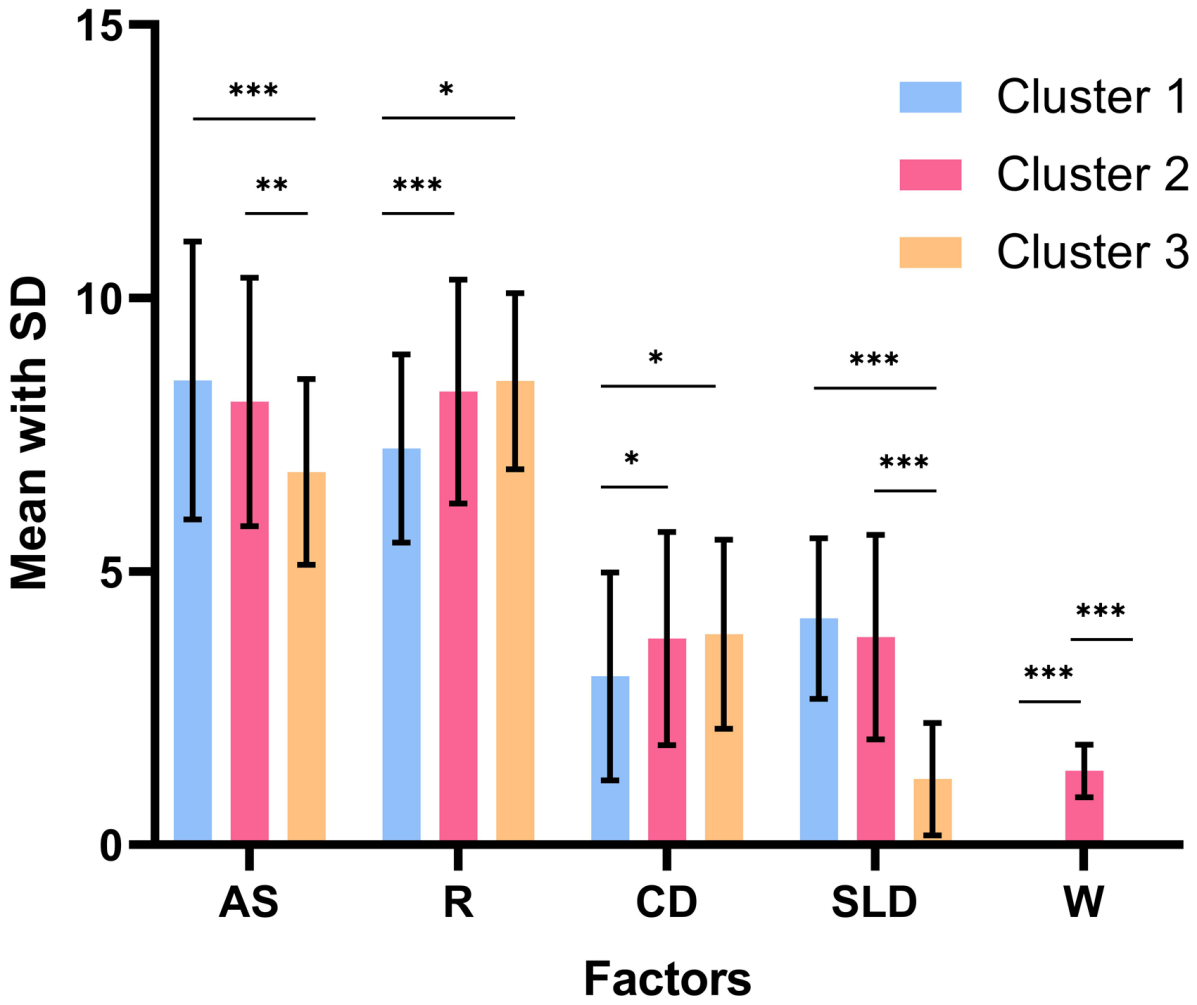
Adjusted for variations in the five symptom dimensions in the three groups. Adjusted for illness duration, BMI, first episode, and antipsychotic drugs. AS, anxiety/somatization; R, retardation; CD, cognitive disturbance; SLD, sleep disruption; W, body weight. ^***^ *p*<0.001, ^**^p < 0.01, and ^*^p < 0.05.

Table 3 presents a comparison of the levels of inflammatory factors in the three clusters. The results of ANOVA revealed no statistically significant differences in these levels. However, ANOVA results for the five dimensions of symptoms indicated significant differences among the three clusters (Table 3; Figure 1), suggesting that there were at least two clusters with statistically significant differences in symptoms. Specifically, significant differences were observed in the anxiety/somatization dimension [*F*(2,273) = 9.673, *p* < 0.001, *η*^2^ = 0.066], retardation dimension, [*F*(2,273) = 11.786, *p* < 0.001, *η*^2^ = 0.079], cognitive disruption dimension [*F*(2,273) = 4.296, *p* = 0.015, *η*^2^ = 0.031], sleep disruption dimension [*F*(2,273) = 65.742, *p* < 0.001, *η*^2^ = 0.325], and body weight dimension [*F*(2,273) = 581.305, *p* < 0.001, *η*^2^ = 0.810]. According to *post hoc* analysis, patients in Cluster 1 (anxiety/somatization: mean ± SD = 8.51 ± 0.24; sleep disruption: mean ± SD = 4.10 ± 0.17) and Cluster 2 (anxiety/somatization: mean ± SD = 8.05 ± 0.21; sleep disruption: mean ± SD = 3.78 ± 0.14) had higher scores on the anxiety/somatization and sleep disruption dimensions compared to those in Cluster 3 (anxiety/somatization: mean ± SD = 6.88 ± 0.29; sleep disruption; mean ± SD = 1.28 ± 0.20). Clusters 2 (mean ± SD = 8.45 ± 0.16) and 3 (mean ± SD = 8.12 ± 0.22) also showed higher scores on the retardation dimension compared to Cluster 1 (mean ± SD = 7.29 ± 0.18) on the retardation dimension. In terms of body weight, Cluster 1 (mean ± SD = 0.003 ± 0.03) and Cluster 3 (mean ± SD = −0.008 ± 0.04) showed less severe symptoms compared to Cluster 2 (mean ± SD =1.36 ± 0.03).

### 3.2. Network structure and centrality measure analysis

**Figure 2:**
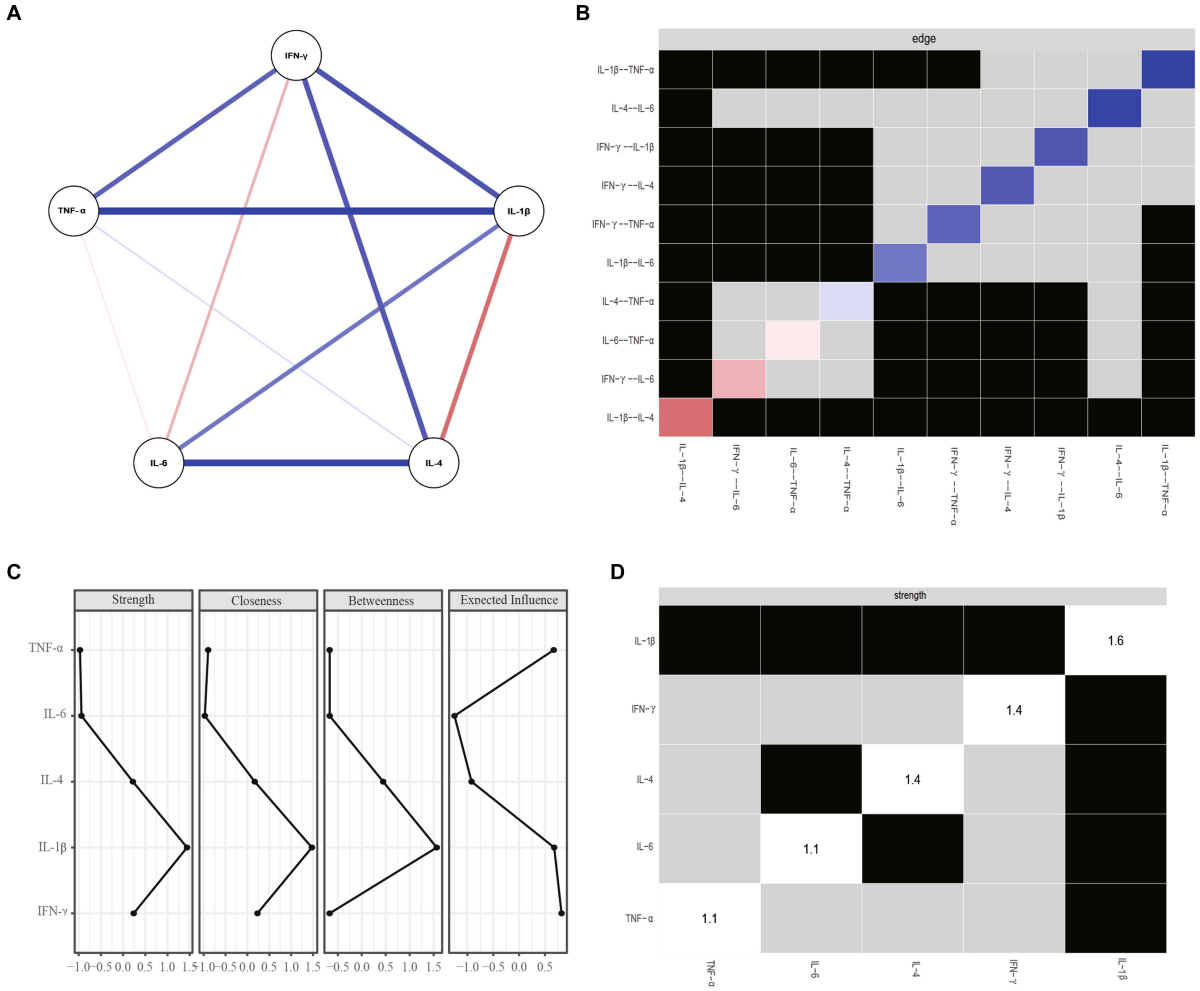
**(A)** Network estimate of overall patients. **(B)** Non-parametric bootstrapped difference test for edges. **(C)** Centrality measures of inflammatory factors within the network of overall patients. **(D)** Nonparametric bootstrapped difference test for strength. Gray boxes show no change between nodes, while black boxes indicate significant difference (*a* = 0.05). Diagonal values indicate node strength. IFN-y, interferon gamma; IL, interleukin; TNF-α, tumor necrosis factor alpha.

Figure 2A shows the network architecture for overall patients with MDD. The network revealed a positive correlation between IL-1β and IL-6 and between TNF-α and IFN-γ, and a negative correlation between IL-4 and IL-1β. Figure 2B shows that node IL-1β had the most important and direct connection with node IL-4.

Figures 2C,D show centrality plots, indicating that IL-1β is the most robust node. TNF-α and IL-4 had significantly higher weights in the network compared to most other inflammatory variables. Therefore, it is crucial to focus on these three inflammatory factors to gain a comprehensive understanding of the role of inflammation in MDD.

### 3.3. Network accuracy and stability

**Figure 3:**
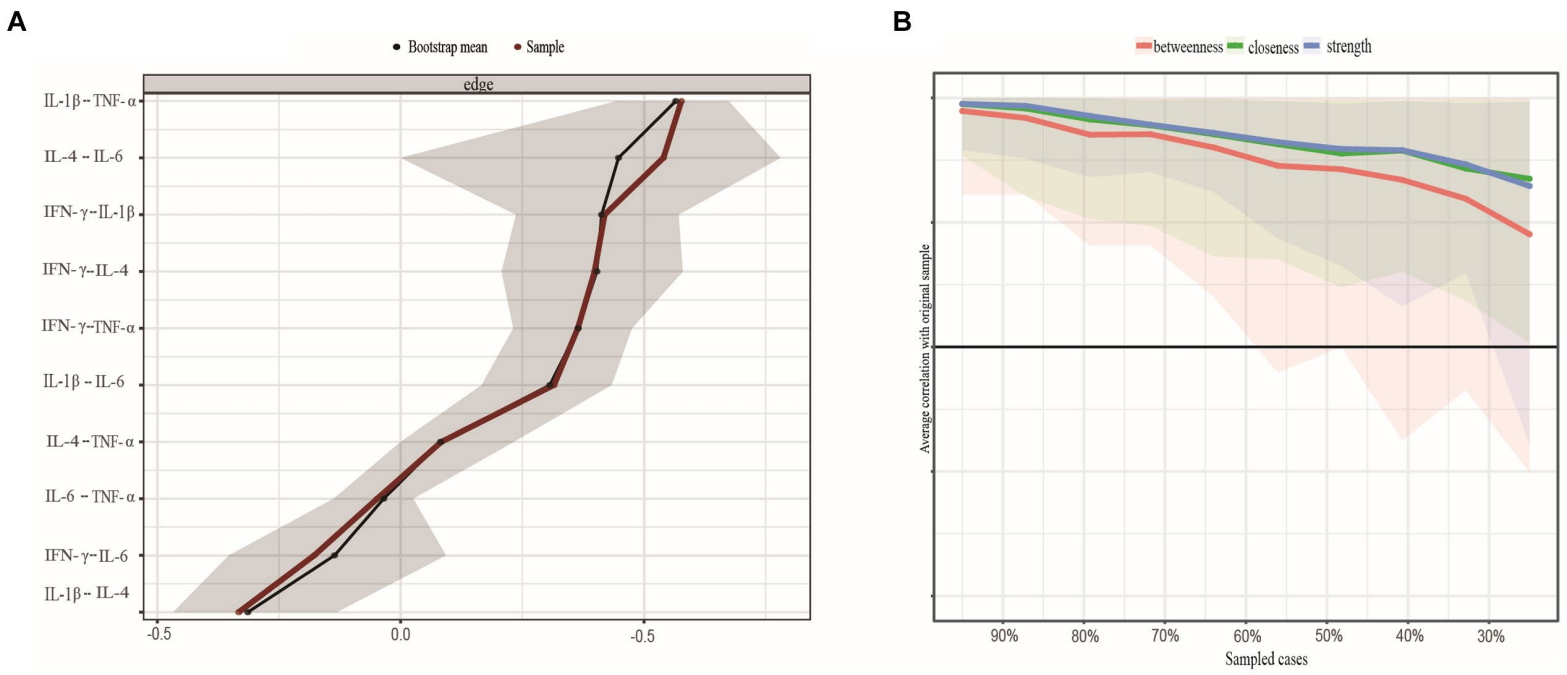
**(A)** Nonparametric bootstrapped confidence intervals of estimated edges. The sample's estimated edge is the red line. 95% bootstrapped confidence interval is grey. **(B)** Stability of centrality indices by case dropping subset bootstrap. Note: Each step's original sample percentage is on the *x*-axis. The *y*-axis shows the average correlation between the centrality indices from the original network and the networks that were re-estimated after deleting increasing percentages of cases. Lines show betweenness, strength, and closeness connections, while areas show 95% CI. IFN-y, interferon gamma; IL, interleukin; TNF-α, tumor necrosis factor alpha.

Nonparametric CI analyses revealed acceptable edge precision for networks constructed with the full dataset (Figure 3A), with smaller CIs indicating more precise edge estimates. We then examined the network’s centrality metrics and the stability of its anticipated influences. Strength (CS-coefficient = 0.361) and expected influence (CS-coefficient = 0.439) reported an acceptable degree of stability, whereas betweenness (CS-coefficient = 0) and closeness (CS-coefficient = 0.207) were less stable (Figure 3B). In other words, the subset-based network center indicator correlated with the initial inflammatory factor central indicator at a 95% confidence level of 0.70 after removing approximately 36% of observations. Overall, the strength index rating of inflammatory factors was credible and accurate.

### 3.4. Network comparison test

**Figure 4:**
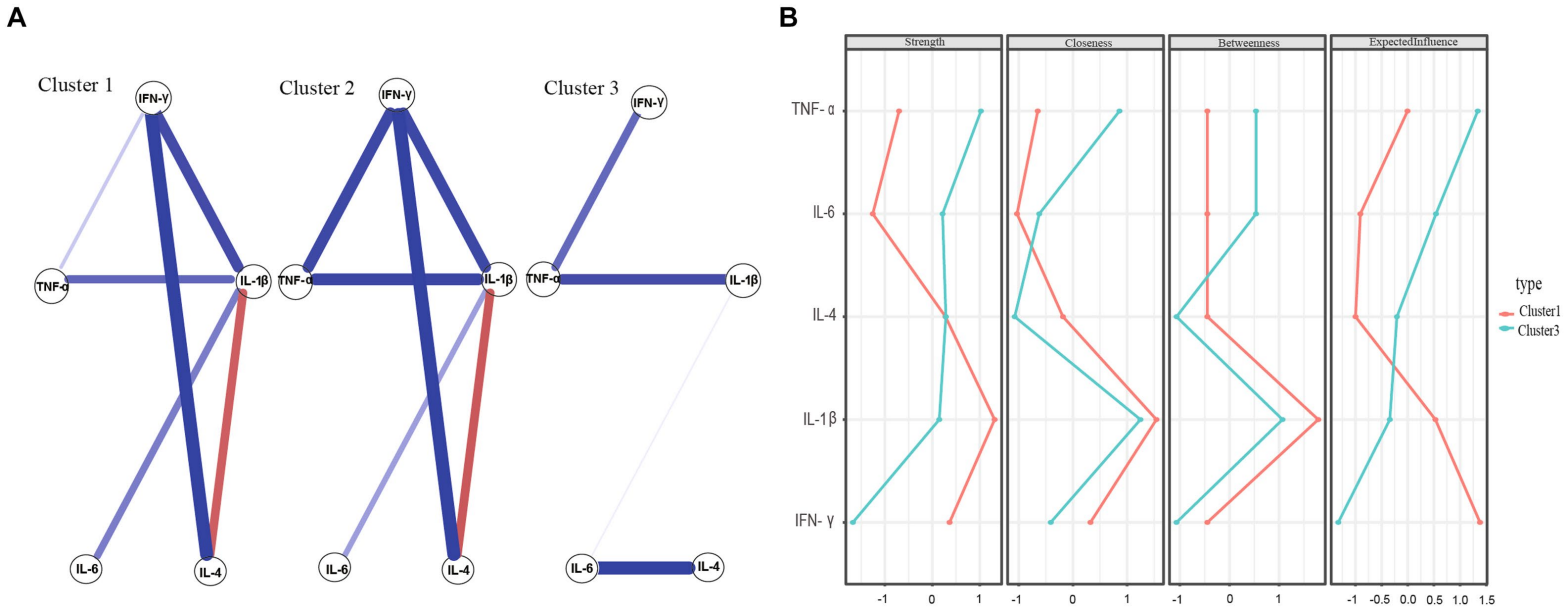
**(A)** Network estimate of three clusters. **(B)** Centrality measures of inflammatory factors within the network of Cluster 1 and Cluster 3.

We controlled for potential confounding variables (i.e., illness duration, BMI, the first episode, and antipsychotic drugs) before evaluating network differences between clusters. Figure 4A shows the networks for Cluster 1, Cluster 2, and Cluster 3. No significant differences were observed between Cluster 1 and Cluster 2 in terms of network structure (*p* = 0.144) or network connectivity. Specifically, the global strength of Cluster 1 (2.906) and Cluster 2 (2.633) did not significantly differ (*p* = 0.558). However, after comparing networks between Cluster 1 and Cluster 3, as well as between Cluster 2 and Cluster 3, we found notable differences in network structure between Cluster 1 and Cluster 3 (*p* < 0.001) and between Cluster 2 and Cluster 3 (*p* = 0.034). Unfortunately, no significant differences were found in network connectivity between Cluster 1 and Cluster 3 (i.e., Cluster 1 global strength = 2.906, Cluster 3 global strength = 2.464, *p* = 0.631) or between Cluster 2 and Cluster 3 (i.e., Cluster 2 global strength = 2.633, Cluster 3 global strength = 2.464, *p* = 0.169).

Figure 4A shows the network estimate of the three clusters. Figure 4B present comparisons of network centricity measures between Clusters 1 and 3 and Clusters 2 and 3, respectively. In terms of strength, IL-1β was the most robust node in both Cluster 1 and Cluster 2 networks, consistent with the outcomes of the entire sample. However, TNF-α was the primary inflammatory factor in the Cluster 3 network. These findings suggest that different subtypes of MDD may be associated with multiple inflammatory markers.

## 4. Discussion

To the best of our knowledge, this is the first study to employ network analysis to investigate inflammatory variables in MDD and their differences in distinct depressive symptom dimensions. The key findings can be summarized as follows: (1) in the inflammatory network of adults with MDD, IL-1β is the most important and central inflammatory factor, and IL-1β and IL-4 are the most closely related inflammatory factors in the overall sample; (2) in Cluster 1 networks, characterized by more severe anxiety/somatization and sleep disruption, IL-1β is the most important inflammatory factor. In Cluster 2 networks, which show more severe anxiety/somatization, sleep disruption, and body weight issues, IL-1β takes on a prominent role. Conversely, in Cluster 3 networks, marked by more severe retardation and cognitive disruption, TNF-α is the most important inflammatory factor.

Pro-inflammatory cytokines have been demonstrated to alter central and neuroendocrine neurotransmitters that respond to stress (39), and IFN-γ immunotherapy has been associated with the development of depression (40). Previous research (41, 42) has also demonstrated that anti-inflammatory medications can reduce depressive symptoms. Based on these findings, our study suggests that IL-1β may be more closely associated with anxiety/somatization and sleep disruption in MDD, whereas TNF-α may be more closely associated with retardation and cognitive disruption, further illuminating the psycho-neuro-inflammatory mechanisms underlying depression. In addition, these findings offer fresh perspectives on the potential treatment of neuropsychiatric conditions, as IL-1β and TNF-α are anticipated to emerge as viable therapeutic targets. Furthermore, these findings may inform future longitudinal and experimental studies aimed at confirming the causal relationship between inflammatory factors and the development of MDD.

Based on the overall strength of the network, IL-1β had the highest centrality index, highlighting its significance in the development of depressive symptoms. This finding is in line with other studies (43), which have also emphasized the importance of IL-1β in neuropsychiatric disorders. With regard to neuropsychiatric illnesses, IL-1β is thought to play a pivotal role as a mediator of various behaviors (44). Elevated plasma levels of IL-1β have been observed in patients with depression, and this cytokine is an efficient stimulator of the hypothalamic–pituitary–adrenal axis (HPA) (45). As IL-1β becomes activated, other immune-associated cells (e.g., monocytes) can produce corresponding immune effects that contribute to an exacerbated inflammatory response (46). In addition, high serum levels of IL-1β stimulate the production of pituitary adrenocorticotropic hormone (ACTH), hypothalamic adrenocorticotropic hormone-releasing hormone (CRH), and adrenal steroids, indirectly leading to depression (47, 48). These findings provide further evidence supporting the role of neuroinflammation in the pathogenesis of MDD.

Animal research (49) also suggests that blocking IL-1β may have therapeutic effects on neuropsychiatric dysfunction. Specifically, studies have shown that deletion of IL-1β in the dentate gyrus (DG) region of the hippocampus significantly reduced depressive and anxious-like behaviors in mice. These findings further support the notion that IL-1β plays a central role in the development of MDD and suggest that IL-1β should be one of the primary targets for future treatments and research on MDD.

Our network analysis also revealed the pivotal role of the association between IL-1β and IL-4 in MDD within the overall sample. The edge connecting IL-1β and IL-4 is the most important edge in the network, suggesting that the link between these two inflammatory factors is a core feature of MDD. Previous studies have shown that increased production of pro-inflammatory cytokines, such as IL-1β and IFN-α, and their enhanced secretion (39) lead to changes in responsive neuroendocrine and central neurotransmitters in response to stress, resulting in various depressive symptoms such as weight loss, general malaise, and sleep disorders (50). Moreover, the literature suggests that depressed patients exhibit reduced levels of anti-inflammatory cytokines such as IL-4 and IL-10 (51, 52). These findings support the notion that an imbalance between pro-inflammatory and anti-inflammatory cytokines plays a significant role in the pathogenesis of MDD.

In line with previous research, a study conducted in rats (53) found that high doses of IL-1β caused behavioral changes and activated the central nervous system inflammatory pathway, whereas IL-4 could counteract the effects of IL-1β on neurotransmitters and central glial cell activation, thereby ameliorating the depressive symptoms caused by IL-1β. IL-4 reduces central and systemic inflammation and reverses IL-1β-induced alterations in neurotransmitter levels. Therefore, our findings, in conjunction with previous discoveries, suggest that the biochemical pathway involving IL-4, an anti-inflammatory factor, and IL-1β, a pro-inflammatory factor, may have therapeutic potential for managing IL-1β-induced depressive behavior However, more research is needed to fully understand the role of the interplay between inflammatory factors in the development of MDD. As the first study to employ networks to examine the inflammatory factor network in MDD, our findings pave the way for further research into the possible mechanisms underlying the involvement of inflammatory factors in this disorder.

After comparing the inflammatory factor networks, we identified statistically significant structural variations between Clusters 1 and 3, as well as between Clusters 2 and 3. Additionally, we discovered some overlap in depressive symptoms between Cluster 1 and Cluster 2. For instance, both Cluster 1 and Cluster 2 exhibited more severe anxiety/somatization and sleep disruption compared to Cluster 3. However, Cluster 3 showed more severe cognitive disruption and retardation compared to Cluster 1, and Cluster 2 had more severe body weight issues compared to Cluster 3. In terms of inflammatory factors, IL-1β was the core inflammatory factor in Clusters 1 and 2, whereas TNF-α was the core inflammatory factor in Cluster 3. We hypothesize that specific depression symptoms are related to distinct inflammatory variables, and IL-1β may be linked to more severe anxiety/somatization, sleep disruption, and body weight issues. Conversely, TNF-α appears to be associated with more severe retardation and cognitive disruption.

As evidence suggests (3), IL-1β may serve as a marker for the initiation of a proinflammatory response specifically targeting anxiety-like behaviors caused by chronic stress, leading to a cascade of inflammatory factor responses. After experiencing repeated social defeat (RSD), the mouse immune system stimulates the HPA axis to produce glucocorticoids, thereby regulating immunity. However, *in vivo*, Ly6Chi monocytes can cause glucocorticoid resistance, which leads to a further increase in the production and release of pro-inflammatory cytokines, such as IL-1β. This action enhances anxiety-like behavior and elevates the expression level of IL-6 in the blood (54). Other studies on animals (55–57) also have demonstrated that upregulation of IL-1β in the brains of murine stress models is associated with the development of anxiety. Moreover, research has indicated that there are high comorbidity rates and longitudinal bidirectional connections between sleep disruption and depression (58). The inflammatory hypothesis has been proposed as a potential mechanism that links sleep disruption with depression (59–61). Various animal experiments, including those on rats (62, 63), rabbits (64), and mice (65), have all demonstrated the somnogenic effects of IL-1β. For example, intraventricular or low-dose intravenous administration of IL-1β has been shown to improve non-rapid eye movement sleep (NREMS), but excessive dosages inhibit sleep (62). A study have suggested that IL-1β may promote sleep, possibly as a feedback regulation following sleep disruption (66). However, in patients with MDD, the ultimate cause of sleep disruption might be damage to this feedback regulation mechanism, leading to persistent sleep disruption and creating a vicious cycle. Another study suggested that IL-1β could decrease 5-hydroxytryptamine (5-HT) concentrations by activating the mitogen-activated protein kinase (67). As is widely recognized, 5-HT is a neurotransmitter that plays a crucial role in regulating various behaviors, including sleep, appetite, aggression, and mood (68). According to the 5-HT hypothesis of depression, the onset of depression is primarily attributed to a decline in 5-HT activity or concentration (69). This can lead to sleep disruption in affected individuals. Thus, sleep disruption, depression, and heightened inflammation are interlinked. Notably, findings from this study indicate that IL-1β is more closely tied to sleep disturbances in patients with MDD than other inflammatory markers. It suggests that IL-1β plays a pivotal role in both the disruption of sleep and the manifestation of depression. While there is no concrete conclusion about alterations in serum inflammatory factors in MDD patients experiencing sleep disturbances, IL-1β seems to be central to this process according to this research. In the future, there may be potential for developing anti-depressant medications that specifically target sleep disorders by focusing on this pathway.

On the other hand, mounting evidence has linked inflammatory processes to depression and obesity (70, 71). Individuals with depression or obesity have been found to exhibit elevated levels of inflammatory biomarkers such as C-reactive protein compared to healthy controls (HCs) (72–74). Our results further imply that IL-1β may be linked to more severe weight issues in patients with MDD. Similarly, numerous animal studies have established a connection between TNF-α and cognitive function. According to early research, TNF-α seems to be essential for normal memory and learning performance in mice under physiological conditions (75). On the other hand, studies involving transgenic mice that overexpress TNF-α have associated with cognitive disruption (76–78). A separate investigation demonstrated that administering TNF-α induced symptoms of depression in mice, including cognitive decline and reduced social engagement (79). An evidence supported that cognitive deterioration linked to TNF-α overexpression may be attributed to abnormalities in synaptic plasticity, which can lead to neurodegeneration (80). These discoveries highlight the complex role that TNF-α plays in cognitive function and mental health, potentially laying the groundwork for further exploration and therapeutic development (81, 82).

Many animal studies have implicated TNF-α in depression; for example, administration of TNF-α has been shown to induce depressive symptoms, such as diminished cognitive performance and reduced social behavior (79). In addition, meta-analyses have shown that plasma levels of TNF-α were higher in individuals with suicidal tendencies than in HCs (83). Together, these data indicate that IL-1β may be more closely linked to anxiety-like mood and sleep disorders, while TNF-α may be linked to depressive mood and cognitive abnormalities, which aligns with our findings.

This study has some limitations that should be acknowledged. Although our study controlled for the first episode of MDD and accounted for the use of antipsychotics as covariates, the specific use of antipsychotics was not documented. Supplementary material S2 showed the dose at which antipsychotics were converted to fluoxetine equivalents. However, current evidence regarding the effect of antipsychotics on cytokine levels remains inconclusive. Some studies have reported that haloperidol can inhibit IFN-γ (84, 85), while others have found enhancement effects (86, 87) or no effect (88). Quetiapine and risperidone exhibit similar uncertain patterns of immunomodulation. In conclusion, there is currently no clear evidence indicating a unidirectional effect of antipsychotics on cytokine levels. It is important to note that different antipsychotics may act as confounding factors when exploring the causal relationship between inflammatory factors and MDD. Future studies should consider the specific type and dosage of antipsychotics to reduce bias and provide a more comprehensive understanding of their impact on inflammatory factors.

In summary, our findings suggest that the future diagnosis and treatment of MDD should consider inflammatory markers in people with depressive symptoms and further explore their association with specific depressive symptoms, especially the effects of IL-1β and TNF-α, and knowledge of the effects of inflammation modification.

## 5. Limitations

Our study had several limitations. First, the present study used a cross-sectional design. Because of this, testing the direction of causation was impossible. To better understand how inflammation contributes to MDD, future studies can use experimental designs or longitudinal methods with extended follow-up periods. Second, although most MDD patients in this study were first-onset and did not take antipsychotics, and the first-onset and current antipsychotic usage were controlled as covariates, future studies will need to discuss potential confounding factors to reduce bias. Third, after the overall sample was clustered, the sample size of each cluster was small, which may have had an impact on the reliability of network comparisons between clusters. Future research needs to recruit more individuals and enlarge the sample size to ensure that the network comparison is accurate.

## 6. Conclusion

In conclusion, our network analysis shows that IL-1β is the core inflammatory factor of MDD and that the IL-1β—IL-4 axis may play an important role in the development of MDD. In addition, network analysis showed that IL-1β was the core inflammatory factor in the population of patients with more severe anxiety/somatization, sleep disruption and body weight. In patients with retardation and cognitive disruption as the main dimensions, TNF-α was the most central factor. The stability and accuracy of these results are reliable, providing a new framework for understanding the inflammatory factors of MDD.

## Data availability statement

The original contributions presented in the study are included in the article/Supplementary material, further inquiries can be directed to the corresponding authors.

## Ethics statement

The study was reviewed and approved by the Institutional Ethics Committee at the Affiliated Brain Hospital of Guangzhou Medical University. All patients completed an informed consent form.

## Author contributions

YN and YaZ designed the study. YaZ recruited participants and collected data. BS, FZ, WL, ZH and XL undertook the statistical analysis. YeZ wrote the first draft of manuscript. BS helped to revise the manuscript. All authors contributed to the article and approved the submitted version.

## Conflict of interest

The authors declare that the research was conducted in the absence of any commercial or financial relationships that could be construed as a potential conflict of interest.

## Publisher’s note

All claims expressed in this article are solely those of the authors and do not necessarily represent those of their affiliated organizations, or those of the publisher, the editors and the reviewers. Any product that may be evaluated in this article, or claim that may be made by its manufacturer, is not guaranteed or endorsed by the publisher.

## Footnotes

^1^ https://vizhub.healthdata.org/gbd-results/
