# Supplementary material for: The core inflammatory factors in patients with major depressive disorder: a network analysis
Source: Front Psychiatry. 2023 Aug 25;14:1216583. doi: 10.3389/fpsyt.2023.1216583 (PMC10491022; doi:10.3389/fpsyt.2023.1216583)
Supplement: Supplementary file 1 [file Data_Sheet_1.PDF]

Supplementary materials

S1 The results of the best model clustering calculation

```
Clustering table:
  1   2   3 
 91 124  65 

Bayesian Information Criterion (BIC):
        EII       VII       EEI       VEI       EVI       VVI       EEE       VEE
1 -5725.171 -5725.171 -5435.746 -5435.746 -5435.746 -5435.746 -5451.562 -5451.562
2 -5676.907 -5679.876 -5351.165 -5306.159 -5414.445 -5420.178 -5429.810 -5435.210
3 -5648.347 -5680.645 -5374.461 -5290.179        NA        NA -5455.392        NA
4 -5643.286 -5679.523 -5417.762 -5290.805        NA        NA -5455.490        NA
5 -5640.833 -5654.731        NA        NA        NA        NA        NA        NA
6 -5658.528 -5654.259        NA        NA        NA        NA        NA        NA
7 -5672.653 -5648.146        NA        NA        NA        NA        NA        NA
8 -5655.903 -5663.289        NA        NA        NA        NA        NA        NA
9 -5673.028 -5667.380        NA        NA        NA        NA        NA        NA
        EVE       VVE       EEV       VEV       EVV       VVV
1 -5451.562 -5451.562 -5451.562 -5451.562 -5451.562 -5451.562
2 -5438.210 -5443.136 -5437.982 -5375.099        NA -5478.630
3        NA        NA -5468.926 -5498.370        NA        NA
4        NA        NA        NA        NA        NA        NA
5        NA        NA        NA        NA        NA        NA
6        NA        NA        NA        NA        NA        NA
7        NA        NA        NA        NA        NA        NA
8        NA        NA        NA        NA        NA        NA
9        NA        NA        NA        NA        NA        NA
```
