# Supplementary material for: The core inflammatory factors in patients with major depressive disorder: a network analysis
Source: Front Psychiatry. 2023 Aug 25;14:1216583. doi: 10.3389/fpsyt.2023.1216583 (PMC10491022; doi:10.3389/fpsyt.2023.1216583)
Supplement: Supplementary file 2 [file Data_Sheet_2.PDF]

Supplementary materials

S2 The antipsychotic-fluoxetine equivalent dose.

Dose (mg)	N (%)
0	200 (71.4)
10.0	7 (2.5)
15.0	3 (1.1)
20.0	28 (10.0)
25.0	1 (0.4)
30.0	6 (2.1)
35.0	1 (0.4)
36.7	1 (0.4)
40.0	6 (2.1)
45.0	3 (1.1)
50.0	1 (0.4)
55.0	1 (0.4)
57.0	1 (0.4)
60.0	14 (5.0)
75.0	1 (0.4)
80.0	5 (1.8)
90.0	1 (0.4)
Total	280 (100)
